# Towards Manufacturing High-Quality Film-Cooling Holes Using Femtosecond Laser Combined with Abrasive Flow

**DOI:** 10.3390/mi16090973

**Published:** 2025-08-25

**Authors:** Lifei Wang, Zhen Wang, Junjie Xu, Wanrong Zhao, Zhen Zhang

**Affiliations:** 1 Science and Technology on Advanced High Temperature Structural Materials Laboratory, Beijing Institute of Aeronautical Materials, Beijing 100095, China; 2 Center for Advanced Laser Technology, School of Electronics and Information Engineering, Hebei University of Technology, Tianjin 300401, China; 3 Hebei Key Laboratory of Advanced Laser Technology and Equipment, Tianjin 300401, China

**Keywords:** femtosecond laser, abrasive flow, film cooling holes, electrical discharge machining

## Abstract

Film-cooling holes are the key cooling structures of turbine blades, and there are still great challenges in manufacturing high-quality film-cooling holes. Although abrasive flow machining can be used as a post-processing technique to optimize the quality of film-cooling holes, its action process and influence mechanism have not been systematically studied. Herein, the drilling method of femtosecond laser combined with abrasive flow is studied in detail. Moreover, for comparison, the drilling methods of single femtosecond laser, single electrical discharge machining, and electrical discharge machining combined with abrasive flow are also discussed. The microstructure and composition distribution of the hole walls before and after abrasive flow machining were systematically characterized, indicating that abrasive flow can effectively remove the recast layer and cause local plastic deformation. Due to the surface hardening and non-uniform residual stress caused by abrasive impact, abrasive flow machining can increase the high-temperature endurance time of film-cooling holes while reducing the elongation. The combination of femtosecond laser and abrasive flow machining demonstrates the best high-temperature mechanical properties, with the endurance time and elongation reaching 136.15 h and 12.1%, respectively. The fracture mechanisms of different drilling methods are further discussed in detail. The research results provide theoretical guidance for the manufacturing of high-quality film-cooling holes through the composite processing of femtosecond laser and abrasive flow.

## 1. Introduction

Turbine blades, as key hot-end components of aircraft engines, operate under extreme conditions of high temperature, high pressure, and high speed, bearing enormous thermal and mechanical stresses [1]. To improve the high-temperature service performance of turbine blades, it is necessary to manufacture a large number of film-cooling holes on the blade body, which can guide the cooling airflow to form a heat-insulating film, thereby increasing the temperature-bearing capacity of the blades by 200–400 K [2,3]. However, the film-cooling holes compromise the structural integrity of the blade, leading to local geometric discontinuities. Stress concentration and thermal defects around the holes can induce crack initiation under thermo-mechanical loads, significantly reducing the high-temperature creep rupture performance of the blades [4,5]. Therefore, there remain significant challenges in how to manufacture high-quality film-cooling holes to ensure the high-temperature performance of the blades.

Femtosecond laser is regarded as the most promising method for manufacturing film-cooling holes due to its extremely small thermal effect and high processing accuracy [6,7]. Relevant scholars have conducted extensive research on the regulation and optimization of the femtosecond laser process. Zhang et al. studied the effects of multidimensional process parameters such as machining route, scanning speed, and power on micro hole taper and roughness, with the focus position being considered to have the greatest impact [8]. High power, positive defocus distance, and small scanning speed are considered the process choices for high-precision and high-quality film-cooling holes. Marimuthu et al. studied the influence of parameters such as pulse width, frequency, and energy on the thickness of the recast layer, and discussed the formation mechanism of the recast layer by numerical simulation and online observation [9]. In our previous study, the effects of laser power, pulse width, and frequency on the thickness of the recast layer, taper, and processing efficiency were also discussed in detail [10]. The above rich and systematic research has provided guidance for optimizing precision and quality in the manufacturing of film-cooling holes. However, quality issues such as thermal stress, recast layer, and large roughness of the hole wall still exist and can accelerate the formation and propagation of microcracks [11,12]. Wang et al. conducted a thermomechanical analysis of the femtosecond laser-drilling process using the finite element method. The thermal stress around the hole increased with the increase in laser power, which easily led to the formation of cracks on the hole wall [13]. Also, Zheng et al. found thermal stress, recast layer, and microcracks in femtosecond laser drilling of Ni-based superalloys with coatings [14]. These defects can lead to fractural failure under the thermal loads of high temperatures and gas impact on the blades, serving as critical factors threatening the service life of the blades.

To optimize the geometric and metallurgical quality of film-cooling holes, the abrasive flow process is introduced for post-treatment [15]. By using abrasive flow to mechanically impact and polish the hole wall, burrs and recast layers can be removed while inducing possible microstructural transformations and residual compressive stresses, which can effectively improve the quality of the hole wall. Zhang et al. employed a hybrid process combining ultrashort pulse laser drilling with abrasive flow machining to fabricate film-cooling holes, followed by a comprehensive analysis of the surface morphology and metallurgical characteristics of the hole walls [16]. The findings demonstrate that abrasive flow machining can effectively eliminate the solidified debris generated during ultrashort pulse laser drilling, significantly reducing surface roughness and inducing a rounded effect at the outlet acute zone of the diffusive hole. Liu et al. investigated the influence of abrasive flow machining on the quality of holes fabricated by electrical discharge machining, and demonstrated that this post-processing technique effectively removes microcracks and recast layers, while markedly reducing surface roughness [17]. However, current research on the combined use of femtosecond laser and abrasive flow processing for drilling film-cooling hole manufacture is relatively limited, particularly with regard to the role of abrasive flow in enhancing the high-temperature mechanical properties of the film-cooling holes remaining unclear.

Herein, the characteristics of film-cooling holes and their high-temperature rupture property under the composite process of femtosecond laser and abrasive flow are systematically studied. A detailed analysis of the fracture mechanism of the target material under thermal–mechanical coupling effects was conducted, and the mechanism by which abrasive flow processing enhances performance was further discussed. Additionally, as a comparison, experiments combining electrical discharge machining (EDM) with abrasive flow processing for drilling are also carried out, and the differences between this method and femtosecond laser processing are discussed. This study provides theoretical guidance for the high-quality manufacturing of film-cooling holes in turbine blades.

## 2. Experimental Methods

The target material is Ni-based superalloy DD6. The nominal chemical composition (ωt%) is as follows: Co 9%, W 8%, Ta 7.5%, Al 5.6%, Cr 4.3%, Re 2%, Mo 2%, Nb 0.5%, Hf 0.1%, Ni balance. Single crystal sample blanks were prepared using directional solidification method, with crystal orientation<001>. After heat treatment, the single crystal sample blanks were machined into flat thin-walled specimens. Holes with a diameter of 0.58 mm were fabricated using EDM and femtosecond laser, positioned at a 60° angle relative to the normal of the thin-walled specimens. A subset of the film-cooling holes was subjected to abrasive flow treatment, and a creep test was conducted at a temperature of 980 °C and a pressure of 300 MPa to obtain the high-temperature mechanical properties. To ensure the reliability of the results and eliminate randomness, 6 replicate tests were conducted for each drilling process. The microstructure and composition distribution of the hole walls were characterized using scanning electron microscopy (SEM) and electron probe X-ray micro analyzer (EPMA).

The femtosecond laser drilling was performed using a commercial infrared laser with a wavelength of 1030 nm and a pulse width of 260 fs. The laser energy distribution in space was Gaussian, with a beam spot size of approximately 50 μm. The femtosecond laser system was equipped with a five-axis numerical control machine tool, which has a total travel range (x, y, z) of 1000 mm × 450 mm × 300 mm. Additionally, an auxiliary gas jet was employed during the femtosecond laser-drilling process to remove the ablation products in real-time. EDM was carried out using a drilling machine produced by DM-CUT. The current was set to 3 A, with a pulse width of 5 μs and a pause duration of 10 μs. During the EDM process, circulating deionized water was used for cooling and flushing away the thermally melted material.

In abrasive flow machining, a dual-vertical-cylinder system is employed to extrude the abrasive medium through the holes. The abrasive flow medium consisted of SiC particles with an average size of approximately 25 μm and a volume fraction of about 30%. The abrasive flow machining was conducted at a pressure of 5 MPa for a duration of 3000 s.

## 3. Results and Discussion

### 3.1. The Microstructure of Film-Cooling Holes

**Figure 1:**
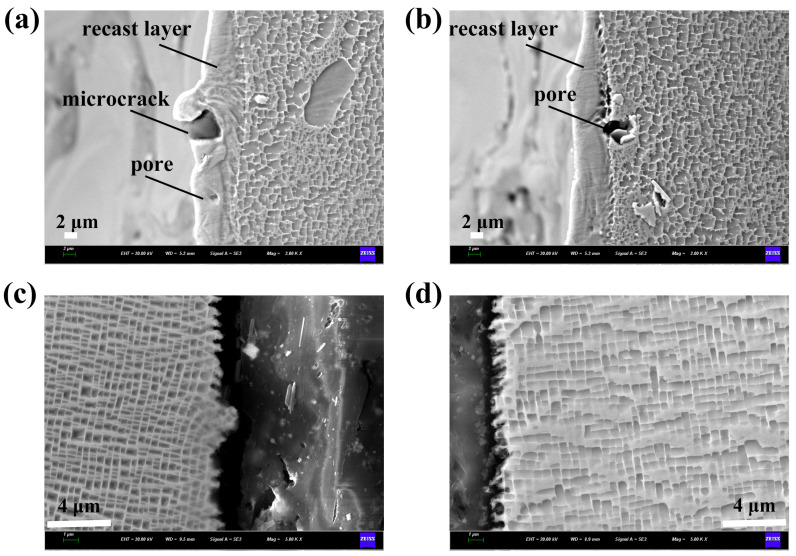
Microstructure of hole walls under different processes. (**a**,**b**) Electric discharge machining, (**c**,**d**) femtosecond laser drilling.

To investigate the characteristics of air film holes after EDM and femtosecond laser processing, the microstructure morphology of the hole walls was systematically characterized, as shown in Figure 1. Obviously, there is a recast layer on the hole wall after EDM, presenting a uniform single-phase structure rather than the grid like biphasic structure of the matrix. During the EDM process, the electrode wire discharges to remove material through thermal melting, and the material is rapidly cooled under the action of deionized water [18,19]. Therefore, it is difficult for the γ’ phase to precipitate for sufficient time. In nickel-based superalloys, the γ′ phase is an ordered L1_2_-structured intermetallic compound that precipitates coherently within the γ matrix. The structure of γ’ phase is usually Ni_3_Al or Ni_3_Ti. Owing to its high thermal stability, low diffusion rate, and strong resistance to dislocation motion, the γ′ phase serves as the primary strengthening mechanism at elevated temperatures. Its volume fraction, size, and distribution play a pivotal role in determining the alloy’s creep resistance, fatigue performance, and overall high-temperature mechanical properties. Additionally, the instability of the melt pool and the Marangoni effect can lead to the flow or even boiling of the molten metal, resulting in the formation of microcracks and gas porosity [20]. The flow of molten metal, driven by temperature gradients and surface tension, also leads to a rippled appearance of the hole walls rather than a smooth surface. In contrast, there is no recast layer on the hole wall after femtosecond laser processing. Under femtosecond laser irradiation, photons are initially coupled with electrons, and the laser energy is swiftly transferred to the electronic system of the target material. Simultaneously, the temperature difference drives the energy transfer from electrons to the lattice, which can be completed within a few picoseconds. However, depending on the thermal conductivity, the thermal conduction between lattices requires tens to hundreds of picoseconds to complete. Therefore, the thermal effect of femtosecond laser processing is extremely limited and does not produce a significant recast layer. It is noteworthy that the hole walls are serrated, which may be due to the uneven removal of the material caused by laser-induced stress waves. The blurred lattice structure observed between the substrate and the serrated region is attributed to thermal effects. Thermal accumulation results in partial heat conduction from the pore wall to the substrate, which is insufficient to cause material melting or ablation. However, this heat can induce intense atomic vibrations, leading to lattice distortion.

**Figure 2:**
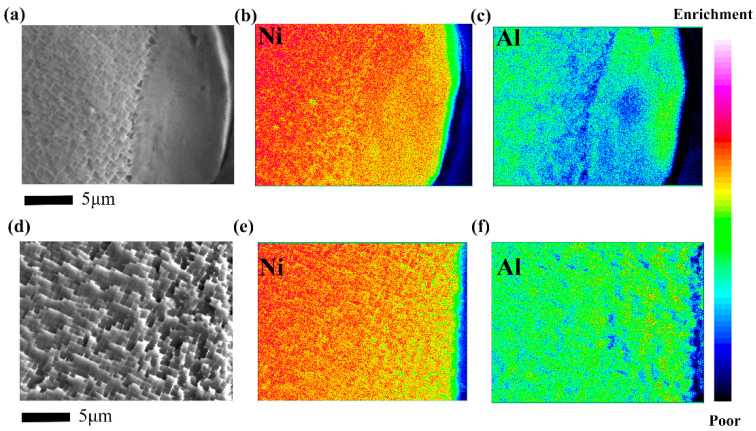
Composition distribution of film cooling hole wall, (**a**–**c**) EDM, (**d**–**f**) femtosecond laser drilling.

Further characterization of the compositional distribution of the hole walls under different processes is shown in Figure 2. For EDM, the content of Al in the recast layer is significantly lower than that in the matrix. In DD6 superalloy, the γ’ strengthening phase typically consists of Ni_3_Al [21]. Under ultrafast heating and cooling conditions, although the diffusion of Al elements is driven by a considerable force, the extremely short duration results in a very limited diffusion distance. The absence of Al element may be another potential reason for the non-precipitation of γ’ phase. In contrast, the element distribution of the hole wall processed by femtosecond laser is consistent with that of the substrate, and there is no obvious segregation.

**Figure 3:**
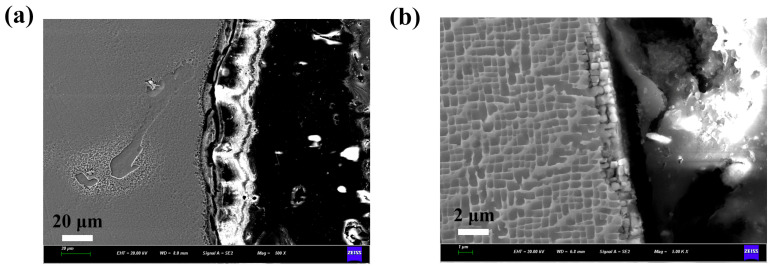
The structure of the film-cooling holes after abrasive flow, (**a**) EDM, (**b**) femtosecond laser drilling.

After abrasive flow machining, the recast layer on the hole walls in electrical discharge machining is mostly removed, leaving only a small number of fragments as shown in Figure 3. During the abrasive flow process, the adhesive abrasive continuously flushes the hole wall, introducing shear stress. Due to the lack of strengthening phase, the hardness and other mechanical properties of the recast layer itself are weaker than those of the matrix. Moreover, internal defects such as microcracks and pores further deteriorate its performance. In this regard, the recast layer is removed by scraping and plowing. The amount of material removed depends on parameters such as abrasive flow pressure, flow rate, and time. For femtosecond laser processing, the serrated morphology of the hole wall is eliminated, and only a small amount of tissue undergoes deformation and torsion. Under higher abrasive flow pressure, abrasive particles impact the hole wall with greater force, resulting in mechanical stress that often exceeds the yield limit of the target material, leading to local plastic deformation. Differences in the initial machining state lead to varying effects of abrasive flow on the microstructure of the film-cooling holes, and the combination of femtosecond laser with abrasive flow achieved the optimal quality.

**Figure 4:**
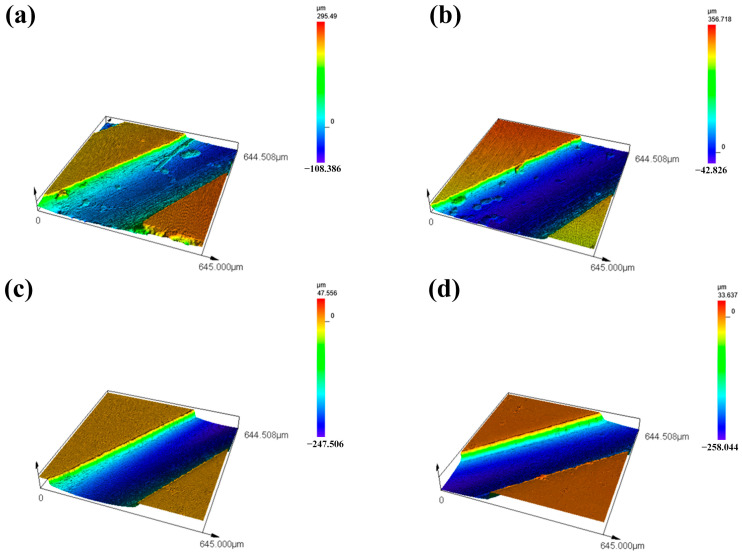
Roughness of hole walls under different processes. (**a**) Electric discharge machining, (**b**) femtosecond laser drilling, (**c**) electric discharge machining and abrasive flow machining, (**d**) femtosecond laser drilling and abrasive flow machining.

Furthermore, as illustrated in Figure 4, the roughness of the hole walls under different processing techniques before and after abrasive flow machining was quantitatively measured using a laser confocal microscope. Prior to abrasive flow machining, both the EDM and femtosecond laser-drilled film-cooling holes exhibited significant roughness on the hole walls, with Ra values of 1.8 μm and 1.2 μm, respectively. In contrast, the abrasive flow effectively removed attachments and protrusions from the hole walls, resulting in significantly smoother surfaces. After abrasive flow machining, the Ra values of the hole walls processed by EDM and femtosecond laser were reduced to 0.6 μm and 0.5 μm, respectively. Therefore, abrasive flow machining exhibits a significant polishing effect on the walls of air film holes.

### 3.2. High-Temperature Creep Property of Film-Cooling Holes

**Figure 5:**
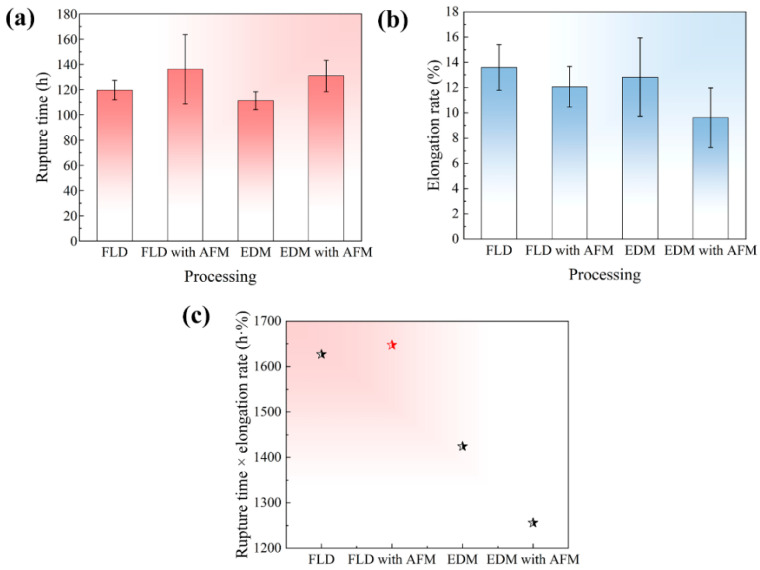
Creep properties under different processes: (**a**) rupture time, (**b**) elongation rate, (**c**) rupture time × elongation rate.

The creep properties under different drilling processes are shown in Figure 5. Before the abrasive flow, the rupture time of the film-cooling holes obtained by femtosecond laser is 119.64 h, which is higher than the 111.26 h obtained by EDM. This difference arises from the recast layer, where microcracks and pores can accelerate the failure. After abrasive flow, the rupture time under different processes has been improved to varying degrees, with femtosecond laser and EDM increasing by 13.8% and 17.59%, respectively, specifically 136.15 h and 130.83 h. Notably, the performance difference between the two drilling processes has been reduced to only 5.32 h. As previously discussed, the abrasive flow almost completely removes the recast layer caused by EDM, making it closer to the film-cooling hole characteristics obtained by femtosecond laser machining. Similarly, the elongation of femtosecond laser machining prior to abrasive flow is slightly higher than that of EDM, with values of 13.6% and 12.8%, respectively. However, the introduction of abrasive flow reduced the elongation, dropping to 12.1% and 9.6%, respectively. During the abrasive flow process, the flow of abrasives typically includes both laminar and turbulent regimes. The different abrasive motion behaviors result in the non-uniform surface hardening and residual stress. Especially, when the abrasive flow velocity is high and the pressure is substantial, elastic turbulence is prone to form at the entrance and exit of the film-cooling holes, and the collision force between the abrasive and the hole wall is much greater than the laminar flow mode of the internal flat hole wall. Therefore, there may be stress concentration near the entrance and exit. Additionally, abrasive collision with the hole wall can cause surface hardening, similar to the shot peening process [22,23]. Both of the above can lead to a decrease in plasticity. In order to evaluate the comprehensive mechanical properties of the film-cooling holes, we multiplied the rupture time by the elongation rate, and the results are shown in Figure 5c. In the high-temperature service of film-cooling holes, it is desirable to achieve an optimal balance between creep rupture life and plastic deformation capacity. The creep rupture life directly reflects the service lifetime of the cooling holes, whereas the plastic deformation capacity indicates their ability to avoid brittle fracture. The maximum product corresponds to the optimal processing route, analogous to the strength-ductility index—defined as the product of tensile strength and elongation—that is widely adopted for evaluating the tensile properties of metallic materials. The drilling mode combining femtosecond laser with abrasive flow exhibits the best comprehensive mechanical properties, specifically with a value of 1647.4 h·%.

It should be noted that the results and conclusions obtained in the present work are based on a single testing condition, and their general applicability remains uncertain. Therefore, additional creep rupture conditions should be investigated in future work, in conjunction with detailed microstructural characterization, to establish more generalizable trends and underlying mechanisms.

### 3.3. The Fracture Mechanism

**Figure 6:**
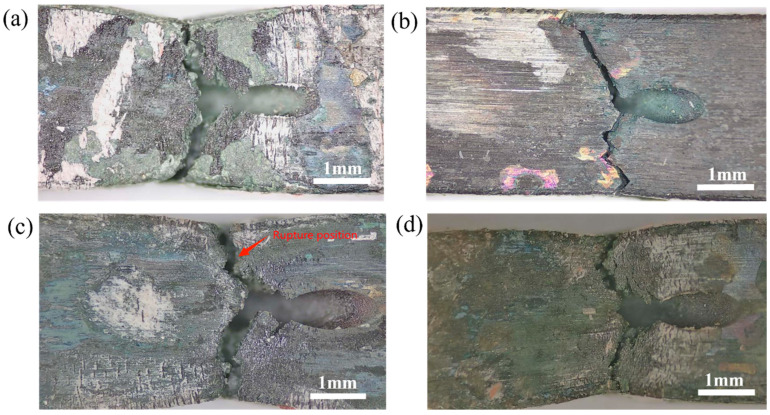
Macroscopic morphology of thin-walled flat specimens after fracture: (**a**) femtosecond laser, (**b**) femtosecond laser-coupled abrasive flow, (**c**) EDM, (**d**) EDM-coupled abrasive flow.

In order to investigate the reasons behind the superiority of the femtosecond laser combined with abrasive flow, the fracture mechanism of film-cooling holes under different processes is further discussed in depth. Figure 6 shows the macroscopic morphology of thin-walled specimens after fracturing under four different processes, with all specimens exhibiting severe oxidation on their surfaces. It is noteworthy that the fracture surface of the film-cooling holes obtained by the combination of femtosecond laser and abrasive flow (Figure 6b) does not show distinct necking, indicating poor plastic deformation capability, which is also the reason for the reduced elongation after the addition of abrasive flow. During creep testing, stress concentration caused by abrasive flow leads to fracture, and the fracture extends parallel to the cross-section of the specimen through the membrane hole connection line, with the overall fracture surface perpendicular to the loading direction. The other three groups of samples exhibited varying degrees of necking, and numerous distinct cracks were observed on their side surfaces.

**Figure 7:**
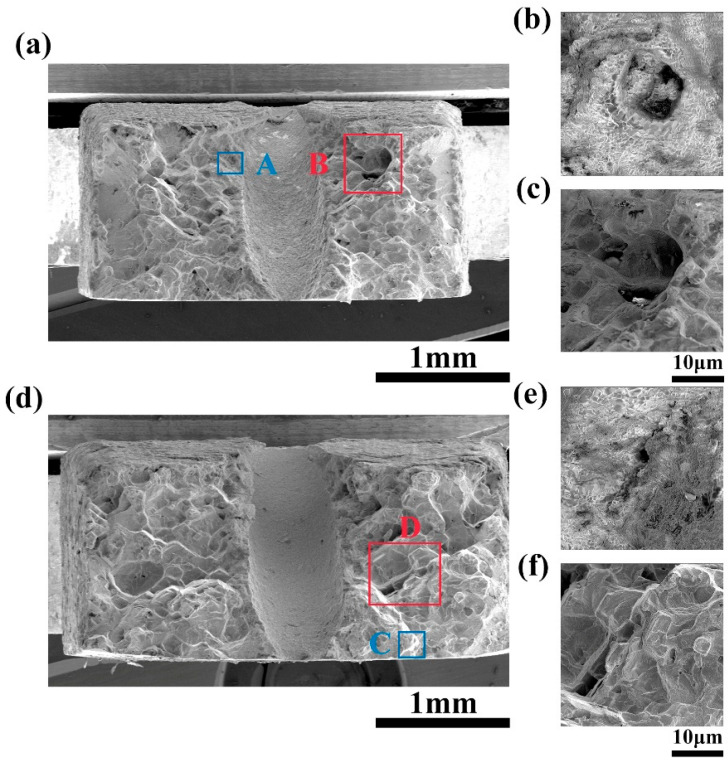
Fracture morphology of EDM and EDM combined with abrasive flow: (**a**) EDM, (**b**) enlarged image of region A, (**c**) enlarged image of region B, (**d**) EDM combined with abrasive flow, (**e**) enlarged image of region C, (**f**) enlarged image of region D.

**Figure 8:**
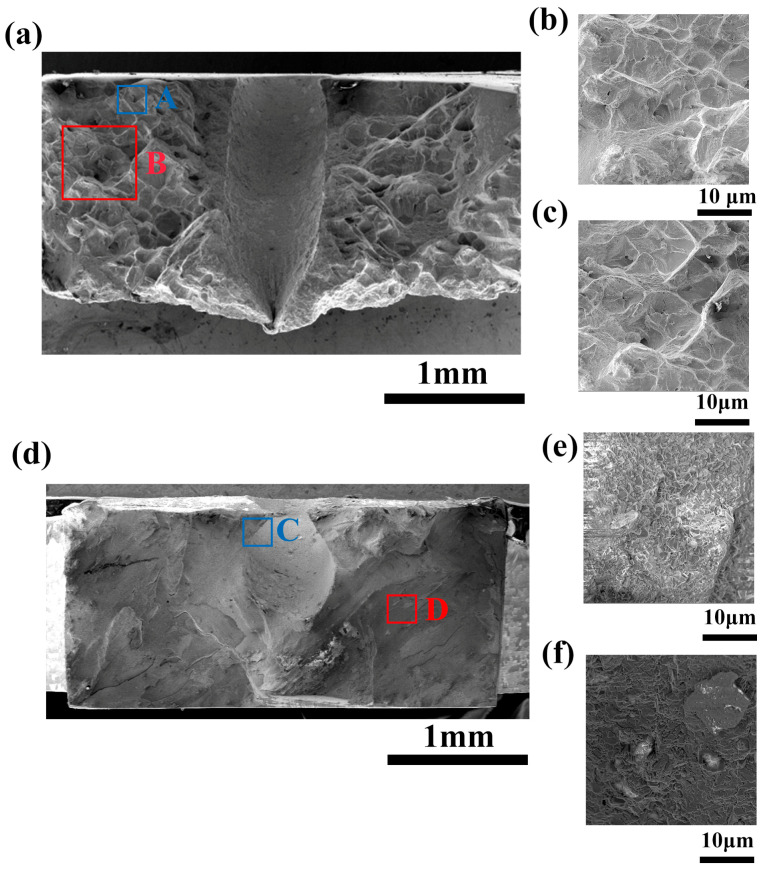
Fracture morphology of femtosecond laser and femtosecond laser combined with abrasive flow: (**a**) femtosecond laser drilling, (**b**) enlarged image of region A, (**c**) enlarged image of region B, (**d**) femtosecond laser combined with abrasive flow, (**e**) enlarged image of region C, (**f**) enlarged image of region D.

The micro fracture morphology is shown in Figure 7 and Figure 8. Under the high-temperature tensile test at 980 °C, severe oxidation occurred on the fracture surfaces of all four samples, while the internal region of the fractures was predominantly characterized by square microfacets. These square surfaces are induced by the growth of microcracks around pre-existing pores within the material, with the facets being connected by dimples or tearing ridges. There are circular holes in the center of the square small plane, which may be pre-existing micropores in the alloy, providing favorable conditions for the initiation and propagation of creep-induced persistent cracks. Once cracks are generated around micropores, they will further propagate at a faster rate, causing cracks to also form around other micropores and ultimately leading to fracture. Thus, the fracture is of a micro-void coalescence type, with porosity and looseness being the primary sources of crack initiation. A large number of crack sources were continuously initiated during the fracture process, resulting in a large number of microcracks, as shown in Figure 6. The differences between the three processes of EDM, EDM-coupled abrasive flow, and femtosecond laser are manifested in the shape and depth of the dimples, as well as the degree of cross-sectional undulations. Unlike the above three methods, the fracture surface of the femtosecond laser-coupled abrasive flow process is smoother, without a large number of square structures and micropores, so there are no cracks in its macroscopic fracture surface (Figure 6b). Therefore, the film-cooling holes obtained by femtosecond laser-coupled abrasive flow exhibit cleavage fracture, with obvious cleavage planes, indicating that the sample underwent minimal plastic deformation before fracture. This fracture mechanism is the reason for its high strength and low elongation, but it achieves the optimal comprehensive performance.

Therefore, considering the microstructure of the initial processing, the microstructure after abrasive flow, and the overall performance, the femtosecond laser-coupled abrasive flow drilling mode is an ideal solution for manufacturing high-quality film-cooling holes.

## 4. Conclusions

In this paper, the drilling method of femtosecond laser composite abrasive flow has been studied in detail, including microstructural changes, high-temperature mechanical properties, and fracture mechanisms. Moreover, single femtosecond laser processing, single EDM, and EDM-combined abrasive flow machining have also been compared and discussed. The main conclusions are as follows:

(1) Femtosecond laser processing does not introduce a recast layer on the film-cooling holes, while EDM results in a recast layer of approximately 2 μm. Different from the matrix, the recast layer has a single-phase γ structure and is deficient in Al element. Moreover, the microcracks, pores, and other defects inside the recast layer are a potential reason for reducing the mechanical properties of the gas film pores. The abrasive flow can almost completely remove the recast layer and cause local plastic deformation on the hole wall.
(2) The high-temperature creep performance of femtosecond laser processing of film-cooling holes is superior to that of EDM, which is caused by the recast layer. The abrasive flow prolongs the rupture time of film-cooling holes but reduces the elongation rate. Specifically, after abrasive flow, the rupture time of the film-cooling holes obtained by femtosecond laser increased from 119.64 h to 136.15 h, an increase of 13.8%. The elongation rate decreased from 13.6% to 12.1%. Based on the comprehensive rupture time and elongation, femtosecond laser combined with abrasive flow machining has the best performance, with a product of 1647.4 h %.
(3) Pore aggregation fracture is the fracture mechanism of film-cooling holes processed by femtosecond laser, EDM, and EDM-combined abrasive flow. The fracture surface is characterized by multiple square regions formed by crack propagation, with porosity and looseness serving as the crack initiation sources. In contrast, the film-cooling holes obtained by femtosecond laser combined with abrasive flow machining exhibit dissociation fractures, and the fracture surface presents a relatively smooth dissociation surface.
(4) The drilling mode of femtosecond laser combined with abrasive flow provides a solution for high-performance film-cooling hole manufacturing and can also be extended to other micro-hole manufacturing fields.

## Data Availability

The data presented in this study are available on request from the corresponding author.
